# Depression in Somatic Disorders: Is There a Beneficial Effect of Exercise?

**DOI:** 10.3389/fpsyt.2019.00141

**Published:** 2019-03-20

**Authors:** Astrid Roeh, Sophie K. Kirchner, Berend Malchow, Isabel Maurus, Andrea Schmitt, Peter Falkai, Alkomiet Hasan

**Affiliations:** ^1^Department of Psychiatry and Psychotherapy, University Hospital, Ludwig-Maximilians University Munich, Munich, Germany; ^2^Department of Psychiatry and Psychotherapy, Universitätsklinikum Jena, Jena, Germany; ^3^Laboratory of Neuroscience (LIM27), Institute of Psychiatry, University of São Paulo, São Paulo, Brazil

**Keywords:** depressive symptoms, training, aerobic, somatic disease, comorbidity

## Abstract

**Background:** The beneficial effects of exercise training on depressive symptoms are well-established. In the past years, more research attention has been drawn to the specific effects of exercise training on depressive symptoms in somatically ill patients. This reviews aims at providing a comprehensive overview of the current findings and evidence of exercise interventions in somatic disorders to improve depressive symptoms.

**Methods:** We systematically searched PubMed and Cochrane databases and extracted meta-analyses from somatically ill patients that underwent exercise interventions and provided information about the outcome of depressive symptoms.

**Results:** Of the 4123 detected publications, 39 were selected for final analysis. Various diseases were included (breast-cancer, prostate cancer, mixed-cancer, cardiovascular disease, coronary heart disease, hemodialysis, fibromyalgia syndrome, acute leukemia, other hematological malignancies, heart failure, HIV, multiple sclerosis, mixed neurological disorders, Parkinson's disease, stroke, ankylosing spondylitis, traumatic brain injury, lupus erythematodes). Most meta-analyses (33/39) found beneficial effects on depressive symptoms, but quality of the included studies as well as duration, intensity, frequency, and type of exercise varied widely.

**Conclusion:** Exercise training has the potential to improve depressive symptoms in patients with somatic disorders. For specific training recommendations, more high quality studies with structured exercise programs and better comparability are needed.

## Introduction

Depression alone and comorbid with other chronic somatic diseases accounts for significant disease burden worldwide (1, 2). By 2030, depression is in addition to HIV/AIDS and ischemic heart disease assumed to be one of the three leading causes of burden of disease (3). Exercise therapy in depressive patients, as an independent intervention and as an adjunct intervention to antidepressant medication or psychotherapy, has been discussed to improve clinical outcomes in somatic diseases (4, 5). Compared to pharmacological treatment, the beneficial effects of exercise seem to be similar and are present at short-term and during follow-up periods of up to one year (6, 7). Especially for somatically unstable patient, this offers new treatment perspectives without the common side-effects of psychopharmacological medication. Usually both the depressive symptoms and the underlying somatic condition improve as a consequence of exercise therapy.

In a large World Health Survey of 245 404 participants from 60 countries, an average between 9.3 and 23.0% of participants with one or more chronic physical diseases had comorbid depressive symptoms (2). For numerous somatic diseases, an association with depression is well-established. For example, patients with multiple sclerosis (8), stroke (9), parkinson's disease (10), diabetes mellitus (11), breast cancer (12) or heart failure (13) have an elevated risk for developing a major depressive disorder (MDD). From a psychiatric perspective, it is important to note that a recently published study with 1,237,194 participants showed that individuals who exercised (various types of disciplines) had 1.49 (43.2%) fewer days of poor mental health in the past month than non-exercising individuals (14).

As the comorbid physical diseases often complicate the pharmacological treatment of depressive symptoms, further research approaches with a focus on alternative therapy options are urgently needed. Based on the beneficial effects of physical exercise in MDD (15), prior studies investigated the effects of exercise in patients with somatic disorders and comorbid depressive symptoms. A meta-analysis of different chronic illnesses including stroke, ischemic heart attack, fibromyalgia, dementia, and other psychiatric diseases, revealed a significant overall reduction of depressive symptoms. The majority of effects were derived from cardiovascular diseases, chronic pain and fibromyalgia (16). The results lead to the conclusion that physical exercise seems to have positive effects on depressive symptoms in patients with various somatic diseases without subdividing the different diseases with specific recommendations. However, a comprehensive overview of the available evidence of how exercise may improve depressive symptoms in patients with somatic diseases is lacking.

In this systematic meta-review, we will provide a detailed overview of recent meta-analyses evaluating different somatic diseases with comorbid depressive symptoms. The efficacy and the types and durations of exercise will be analyzed. Up to date, meta-analyses of specific diseases or disease groups (e.g., cardiovascular diseases) have been published as well as meta-analyses comprising different somatic diseases with an emphasis on cardiovascular diseases, fibromyalgia and pain (16). With our review, we aim to close this gap and point to limitations of prior studies as well as possible future research strategies.

## Methods

We systematically searched PubMed and Cochrane databases with all combinations of the following search terms: depressive disorder OR depression AND exercise, depressive disorder OR depression AND physical activity, depressive disorder OR depression AND endurance training, depressive disorder OR depression AND training, depressive disorder OR depression AND resistance training, depressive disorder OR depression AND aerobic, depression OR depressive disorder AND somatic AND exercise, depression OR depressive disorder AND physical illness. The database search was last updated on 31th July 2018. All citations were screened for relevance by title in a first step, by abstract in a second step and by full-text in a last step. In all included meta-analyses, the citations were screened manually for further relevant meta-analyses that may have not been detected by the systematic search. The systematic literature search and selection was performed by AR, the selection was afterwards reviewed independently by SK.

Both AR and SK retrieved the relevant information and the results were compared. In case of disagreement, a third author (AH) was consulted. Inclusion criteria were: meta-analysis investigating the effects of exercise on depressive symptoms in patients with comorbid physical illness based on interventional trials, published within the past 10 years. Exclusion criteria contained meta-analyses published earlier than within the past 10 years, systematic reviews without meta-analyses, meta-analyses in non-English language, meta-analyses only investigating depressive patients with no somatic comorbidities, lack of data post-intervention. No limitations were defined for the type or duration of exercise and the type of somatic illness.

## Results

### Study Selection

**Figure 1:**
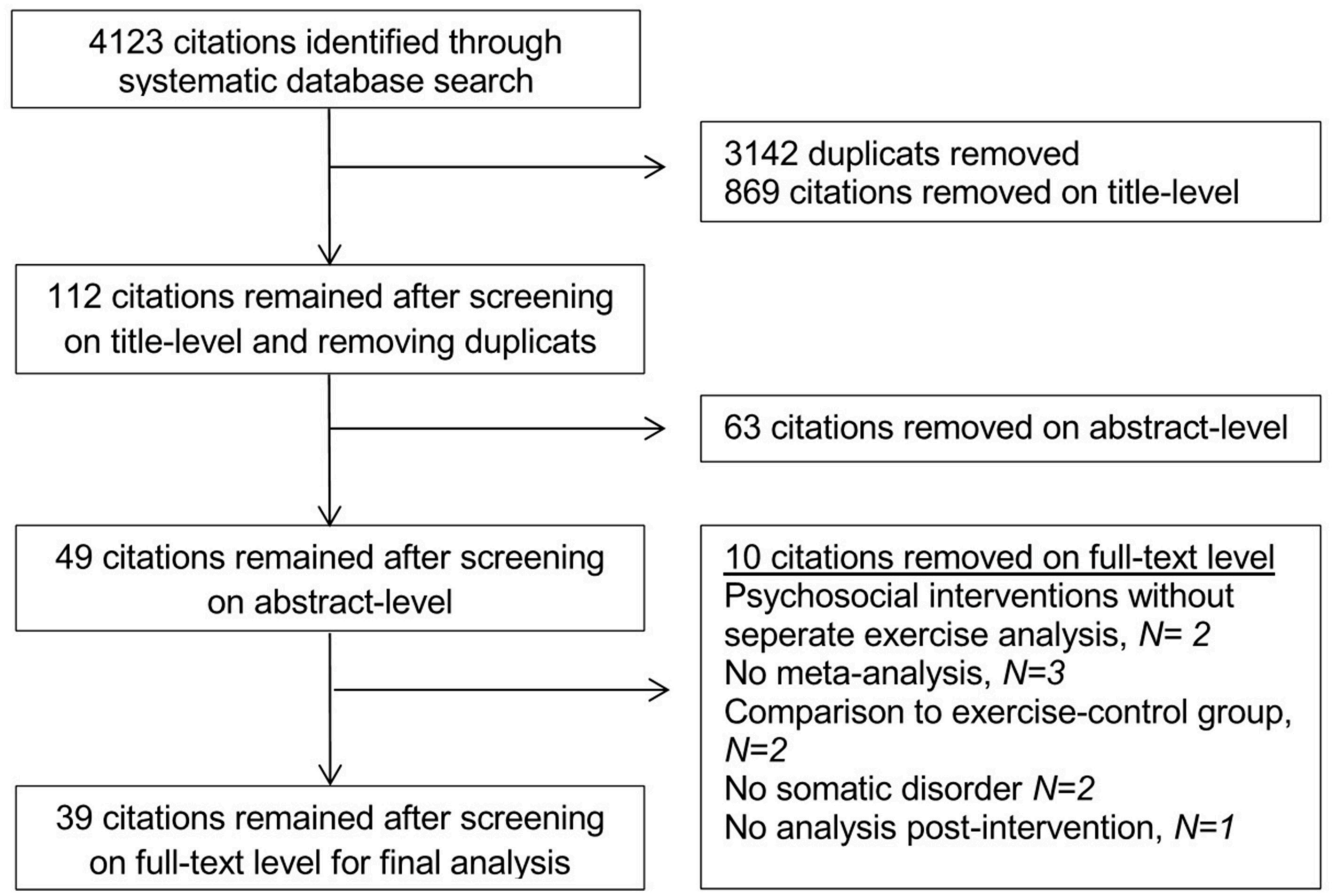
Flow-chart of included citations.

The initial search without further restrictions resulted in 103,468 citations. After limiting the citations for the past 10 years, 60,430 citations remained. When only considering the meta-analyses, 4,123 citations remained and after eliminating duplicates, 981 citations were included for further analysis. The screening on title-level eliminated 869 citations (112 remaining), the screening on abstract-level another 63 citations (49 remaining). After full-text screening, 39 citations remained for final analysis (see Figure 1).

### Study Characteristics

Table 1 provides an overview of the included somatic diseases. The duration of the single studies varied between three (46) to 52 (21) weeks, the intensity between one time (51) and seven times (37) per week and the duration of the sessions between 30 and 122 min (16). Adherence rate was solely reported in two meta-analyses (21, 22) and intensity of the training with heart rate controlled exertion in one meta-analysis (37). The interventions consisted of aerobic exercise, resistance training, balance training, yoga, moderate cycling, walking, nordic walking, running, swimming, tai chi chuan, qigong, Bobath exercises, jogging, calisthenics. Across all trials, the mean sample size was 658 with a minimum of 39 (28) and a maximum of 3.425 (36). Depressive symptoms were rated with different questionnaires (observer or self-assessment): BDI (Beck Depression Inventory), CDI (Children Depression Inventory), CES-D (Center for Epidemiological Studies Depression Scale), DASS (Depression Anxiety Stress Scales), GDS (Geriatric Depression Scale), HAD (Hospital Anxiety and Depression Scale), HAM-D (Hamilton Rating Scale for Depression), SCL-90 (Symptom Checklist-90), TAS (Toronto Attitude Scale), POMS (Profile of Mood States), SAS (Symptom Assessment Scale), CSDD (Cornell Scale for Depression in Dementia), IDS-SR (Inventory of Depressive Symptomatology Self Report), LPD (Levine-Pilowsky Depression Questionnaire), MADRS (Montgomery-Asberg Depression Rating Scale), MDI (Major Depression Inventory), POMS (Profile of Mood States), QOL (Quality of Life), DASS (Depression Anxiety Stress Scales), TAS (Toronto Attitude Scale), MAACL (Multiple Affect Adjective Checklist), HDCDS (Hare-Davis Cardiac Depression Scale), SDS (Self-rating Depression Scale), BSI-18 (Brief Symptom Inventory). Thus, in the different meta-analyses, various different questionnaires were used, most often self-rating questionnaires: the BDI I/II in 26, the HADS in 18 and the CES-D in 17 meta-analyses. The majority of meta-analyses included more than three different questionnaires. As observer-rating scales, HAMD was used in four and MADRS in three meta-analyses.

**Table 1:** Included somatic diseases.

**Somatic disease in meta-analysis**	**No. of meta-analyses**	**References**
Breast cancer	7	(17–23)
Mixed cancer types	7	(24–30)
Prostate cancer	3	(31–33)
Cardiovascular disease	1	(34)
Coronary Heart disease	1	(35)
Heart failure	2	(36, 37)
Intradialytic patients	2	(38, 39)
Hemodialysis patients	1	(40)
Fibromyalgia	2	(41, 42)
Ankylosing spondylitis	1	(43)
Lupus erythematodes	1	(44)
Acute leukemia	1	(45)
Hematological malignancies	1	(46)
HIV	1	(47)
Multiple sclerosis	3	(16, 48, 49)
Mixed neurological disorders	1	(50)
Parkinson disease	1	(51)
Stroke	2	(52, 53)
Traumatic brain injury	1	(54)
Total	39	

*HIV, human immunodeficiency virus*.

### Risk of Bias Within and Across Studies

Most meta-analyses (51) reported about different cancer-type patients followed by neurological disorders (7). For other diseases, such as HIV, only one meta-analysis could be identified. The variety of exercise modalities and the different durations of interventions lead to a reduced general transferability of the conclusions. Recommendations regarding effects of specific interventions on depressive symptoms in specific somatic conditions can hardly be drawn. None of the trials included in the respective meta-analyses could be declared double-blind as this is challenging to fulfill with exercise interventions. The application of various different rating-scales is another potential risk of bias. Moreover, most of the included studies in the respective meta-analyses did not define depressive symptoms as primary outcome parameter.

### Results of Individual Studies

A detailed summary of the included studies with inclusion/exclusion criteria, study population, intervention summary and outcomes is provided in Tables 2, 3.

**Table 2:** Methods and Limitations of the included meta-analyses.

**References**	**Included trials, sample size**	**Included population/inclusion criteria**	**Intervention**	**Rating Scales**	**Quality and possible moderators**
Adamson et al. (50)	23 studies, *N* = 1.324 participants	1) Adults aged >18 years 2) Any diagnosed neurologic disorder (ad or dementia, migraine, ms, parkinson disease, spinal cord injury, and traumatic brain injury) Excluded: fibromyalgia, rheumatoid arthritis	Aerobic exercise, resistance training, balance training, yoga, and others involving a combination of these exercises. No limits concerning frequency, intensity, or duration of the exercise intervention.	BDI, BDI-II, CES-D, CSDD, GDS, HAD, IDS-SR, MADRS, LPD, MDI, POMS	123/26 studies evaluated depression. 13 of the 26 studies Received a score >6 on the PEDro scale. 3 trials identified depression as primary endpoint.
Bergenthal et al. (46)	3 RCTs, *N* = 249	1) RCTs comparing an aerobic physical exercise intervention, intending to improve the oxygen system, in addition to standard care with standard care 2) Only for adults suffering from hematological malignancies.	Studies that evaluated aerobic exercise (such as moderate cycling, walking, Nordic walking, running, swimming and other related forms of sport) or aerobic exercise in addition to strength training were included. Studies that investigated the effect of training programs that were composed of yoga, tai chi chuan, qigong or similar types of exercise were excluded. Duration of the intervention between 3 and 12 weeks with 3–5 sessions per week.	NR	3/9 studies could be included in the meta-analysis for depression (secondary endpoint). Low quality of evidence.
Brown et al. (24)	37 RCTs, *N* = 2.929	1) RCT comparing an exercise intervention with a control group 2) Report of depression outcomes 3) Adults diagnosed with any type of cancer, regardless of stage of diagnosis or type or stage of treatment At baseline, the standardized metric of depressive symptoms was 34.2 and ranged from 3.49 to 81.5. 65% Breast Cancer, 5% Prostate cancer, 5% Leukemia, 5% Lymphoma, 20% diverse groups of cancer	Exercise interventions occurring in any setting, with or without supervision. Mean length of the exercise interventions was 13.2 weeks with an average of 3.0 sessions per week lasting 49.1 min/session.	CES-D, POMS, BDI, HAS, SAS	The mean PEDro score of the exercise interventions was 7.061 suggesting relatively high methodological quality.
Buffart et al. (25)	8 RCTs, *N* = 471	1) Design: RCT 2) Population: adults with any cancer diagnosis either during or post treatment 3) Intervention: yoga including physical postures (asanas) 4) Control group: non-exercise or wait-list 5) Outcome: physical and psychosocial outcomes Average age of the participants ranged from 44 to 63 years. 1 study with lymphoma patients, the rest with breast cancer patients.	All included a supervised yoga program with physical poses (yoga asanas), combined with breathing techniques (pranayama) and relaxation or meditation (savasana or dhanya). Median program duration was 7 weeks with a range of 6 weeks to 6 months. In general, the number of classes per week ranged from one to three, and home practice was encouraged in nine studies, supported by audio or videotapes. Session duration ranged from 30 to 120 min; three studies did not report the session duration.	HADS-D, BDI, CES-D, POMS	The median quality score was 67% (range: 22–89%). All but one study were of high quality. 8 of 16 studies evaluated depression.
Carayol et al. (17)	9 RCTs, *N* = 801	1) Participants were adult women diagnosed with breast cancer, 2) Presented a randomized, controlled experimental design 3) Included an intervention program involving physical activity (yoga-based interventions were included whereas relaxation-based were not) 4) Intervention program was scheduled during adjuvant cancer therapy (chemotherapy and/or radiotherapy), (v) assessed at least one psychological outcome among fatigue, anxiety, depression, and QoL 5) Provided pre and post-intervention data to calculate standardized mean differences(SMDs) The median age of included patients was 50.5 years old. All patients with breast cancer have been diagnosed with non-metastatic cancer and were undergoing adjuvant therapy, i.e., chemotherapy and/or radiotherapy during exercise intervention.	Exercise duration between 5 and 26 weeks, 2–6 sessions per week of 30–60 min. Interventions consisted of aerobic exercise and/or resistance training or Yoga.	CES-D, HADS-D, BDI, POMS	Regarding methodological quality, median score was 7, ranging from 2 to 9. 9/17 studies evaluated depression.
Chung et al. (38)	4 RCTs, *N* = 212	1) This review included randomized controlled trials (RCTs) published in the English or Chinese language 2) participants aged >18 years diagnosed with end stage renal disease (ESRD) requiring maintenance hemodialysis (HD) as renal replacement therapy 3) participants undergoing HD for more than 3 months who received exercise training during HD sessions (intradialytic)	Exercise interventions included aerobic cycling alone, resistance training, cycling or resistance training and aerobic cycling combined with strength training or range of motion. Each exercise session lasted 10–90 min. The exercise program lasted between 8 and 48 weeks.	Zung Depression Scale, BDI	In over half of the studies, there was a low or unclear risk of detection bias. 4/17 studies evaluated depression.
Craft et al. (26)	14 RCTs, *N* = 1.371 participants	1) RCTs of adults diagnosed with cancer 2) Comparison of an exercise program with usual care 3) The exercise program was chronic in nature (i.e., at least 4 weeks in duration) 4) Reported depressive symptoms pre- and post-intervention 5) Utilized a depression inventory or a clinician interview to quantify depressive symptoms 6) published in English Average age of participants was 51.6 years. 9 (60%) breast cancer, 1 colorectal cancer, 1 lymphoma, 2 prostate cancer and 2 diverse groups of cancer	All studies included an aerobic exercise component, with several also including a strength training component.	CES-D, BDI, HADS, QOL	Only 1 trial identified depression as primary endpoint. 11 RCTs with PEDro “high” quality. Potential moderators: exercise location, Supervised and partially supervised exercise, exercise bout durations of >30 min.
Cramer et al. (19)	6 RCTs, *N* = 161	1) RCTs 2) Studies of adult (older than 18 years) patients with a history of breast cancer 3) Studies that compared yoga with no treatment or any active treatment 4) No restrictions were made regarding yoga tradition, length, frequency or duration of the program. Co-interventions were allowed. 5) Studies that assessed health-related quality of life or well-being and/or psychological health.	Yoga interventions were heterogeneous. Program length and intensity varied, ranging from daily interventions over 1 week to one intervention per week over 6 months.	CES-D, HADS, BDI	Generally, risk of selection bias was high. 6/10 studies evaluated depression.
Cramer et al. (18)	11 RCTs, *N* = 496 (7 RCTs yoga vs. No therapy) + *N* = 226 (4 RCTs, yoga vs. Psychosocial intervention)	1) RCT 2) Compared yoga interventions vs. no therapy or vs. any other active therapy in women with a diagnosis of non-metastatic or metastatic breast cancer 3) Assessed at least one of the primary outcomes on patient-reported instruments, including health-related quality of life, depression, anxiety, fatigue or sleep disturbances	Of the 24 included studies: the duration of yoga programs ranged from 2 weeks to 6 months, with a median duration of 8 weeks; the frequency of yoga interventions ranged from one to 10 (median: two) weekly yoga sessions of 20 to 120 (median: 67.5) min in length.	HADS, BDI, CES-D, POMS	11/24 studies evaluated depression.
Dalgas et al. (48)	12 RCTs, *N* = 476	1) RCT design 2) Enrollment of participants with definite MS according to the McDonald criteria 3) Evaluate an exercise intervention that was compared with either non-training controls, active controls or another exercise intervention 4) Inclusion of a primary validated measure of depressive symptoms available in English	resistance training, endurance training, combined training (i.e., resistance training + endurance training) or as other exercise modalities	BDI, MDI, IDS-SR, HADS, POMS, CES-D	Only one of the RCT studies applied depressive symptoms As the primary outcome. Average PEDro score was 5.6 ± 1.3 points.
Eng and Reime (52)	13 RCTs, *N* = 1.022	1) Confirmed diagnosis of stroke by medical records, imaging, or clinical examination 2) Adult patients over 18 years of age 3) Intervention and control group treatments clearly defined 4) Baseline and follow-up of at least 4 weeks available for depressive symptoms The time since stroke ranged from 30 days to 6 years and the patients' age ranged from 21 to 93 years.	Progressive resistance training, functional, aerobic exercises, treadmill exercises, Bobath exercises, individualized exercises with education, community-based rehabilitation services including physical therapy and occupational therapy. Eleven of the 13 studies had a frequency of at least two sessions per week for a minimal duration of 4 weeks. The length of the intervention ranged from 4 weeks to 12 weeks.	HAD, GDS, BDI, CES-D	PEDro scale ranged from three to eight.
		The mean baseline depressive symptoms of the study samples were below established thresholds for clinically relevant depressive symptoms in the majority of studies (12/13).			
Ensari et al. (49)	13 RCTs, *N* = 477	1)Studies that compared exercise training vs. no-treatment control Reliable and valid measures of depressive symptoms (e.g., HADS, CES-D) as an outcome assessment pre/post intervention in patients with MS2) Only samples in 2 of the studies had mean scores above the threshold for moderate depressive symptomatology.	Aerobic and an-aerobic. 7 of the 13 studies had a frequency of at least 3 sessions per week.	BDI, IDS, MDI, HADS, CES-D, POMS	9 of the 13 studies received a score of 6 or higher on the PEDro scale. Exploratory analyses only identified depression symptom scale as a potential moderator variable (*p* = 0.04).
Fong et al. (27)	4 RCTs, *N* = 168 4 RCTs with different outcome measurements, *N* = 533	Adult patients (aged ≥18) Patients diagnosed with cancer3) Patients who had completed their main treatment for cancer but might be still undergoing hormonal treatment4) And assessed the effect of physical activity on health indicators $\frac{3}{4}$ included studies evaluated breast cancer, 1 endometrial cancer.	All 4 studies used aerobic exercise.	BDI, HADS, POMS	4/34 studies evaluated depression via BDI and were included in the meta-analysis. 2 studies used the HADS and 2 the POMS. No overall analysis was performed.
Furmaniak et al. (20)	5 RCTs, *N* = 674	1) RCTs of exercise training during adjuvant (including neoadjuvant) treatment (radiotherapy chemotherapy) for women with non-metastatic breast cancer 2) Exercise training during adjuvant (including neoadjuvant) treatment (radiotherapy, chemotherapy) for women with non-metastatic breast cancer. 3) Studies that assessed the effects of all forms of repeatedly performed aerobic or resistance exercise or both with program duration of at least 6 weeks	Aerobic or resistance training.	BDI, CES-D	Cochranes risk of bias tool. 1 study evaluated depression via HADS and was not included in the meta-analysis. 5/32 studies evaluated depression.
Gomes Neto et al. (39)	3 RCTs, *N* = 88	1) Studies that included hemodialysis patients randomized to two different intra-dialytic exercise training modalities or to a group of specific exercise modality and group of usual care without exercise training. 2) Studies that enrolled patients with cardiac or respiratory diseases were excluded	Combined aerobic and resistance training.	BDI	3/56 studies evaluated depression with the BDI.
Graven et al. (53)	54 RCTs, 10 with exercise and 2 for meta-analysis, *N* = 137	1) Patients with a primary diagnosis of cerebrovascular accident 2) Participants who were community-dwelling 3) RCTs 4) Trial outcomes that measured at least one of the domains of participation, the mood disorder of depression and HRQoL (using validated scales that are commonly applied) 5) Interventions conducted in the community setting by predominantly nursing or allied health practitioners 6) The availability of an English full text version	All the studies incorporated an experimental exercise regime that was of an intensity of two to three sessions per week over a 6–12-week duration (average 9.8 weeks).	CES-D, GDS-15	PEDro methodological rating of at least four points was an inclusion criterion.
Herring et al. (16)	14 RCTs, *N* = 624	1) English-language peer-reviewed publications 2) Adults aged >18 years with a formal diagnosis of MS 3) Randomized allocation to either an exercise intervention or a non-active control condition that lacked exercise training 4) A measure of depressive symptoms measured at baseline and at mid- and/or post-intervention The mean age was 44.0 years. The mean percentage of women was 75%. Mean reported disease duration was 9.8 years, and mean baseline Expanded Disability Status Scale (EDSS) score was 3.4.	Aerobic, Resistance Aerobic+, Resistance+, Yoga Exercise training consisted of three sessions per week, 51 min per session, and 11 weeks in duration. Based on reported methods, PwMS were prescribed 122 (*SD* = 38) min of exercise per week. The mean exercise training adherence rate was 85%.	BDI, CES-D, HADS-D, IDS-SR, POMS-D, MDI	Mean PEDro score was 5.86. Depressive symptoms were not the primary outcome in any of the included trials. Langhorst et al. (41)	6 RCTs, *N* = 306	1) RCTs 2) Studies with meditative movement therapies 3) Patients diagnosed with FMS on recognized criteria, of any age 4) Studies should assess at least one key domain of FMS (pain, sleep, fatigue), health-related quality of life (HRQOL) and depression	Tai Chi and/or Yoga, and/or Qi Gong. In the whole review (7 studies), the number of sessions was 12 (8–24). The total treatment time was 18 (6–48) h.	BDI, CES-D, CDI	Publication bias was assessed by Egger's intercept test and Begg's rank correlation test at the significance level *p* < 0.05. 6/7 studies evaluated depression.
Liang et al. (43)	3 RCTs/quasi-RCTs, *N* = 121	1) Adults diagnosed by a rheumatologist as having AS (ankylosing spondylitis) 2) Participants > 18 years 3) Quasi-randomized and randomized controlled trials (RCT), in which at least one of the groups received home-based exercise therapy	Home-based exercise program including muscle relaxation, flexibility exercises for cervical, thoracic and lumbar spine, range of motion exercises of coxofemoral joints, stretching exercises for the major muscle groups, muscular strengthening, straight posture, and respiratory exercises.	BDI	3/6 trials investigated depression. The research team performed an analysis of all included studies (6), using a funnel plot to determine publication bias in all the literature. The outcome from the funnel shows asymmetry, thereby indicating that publication bias possibly exists in the included studies.
Lin et al. (29)	8 RCTs, *N* = 519	1) Randomized control trial design 2) Examination of yoga or MBSR (mindfulness based stress reduction) on psychological health, quality of life, and physical health of cancer patients 6 studies assessed the effects of yoga for patients with breast cancer, 1 for patients with lymphoma, and 1 for mixed cancer population.	The style of yoga used and the duration and frequency of the yoga sessions varied among all studies. Intervention duration ranged from 6 to 24 weeks.	HADS, CES-D, POMS SCL-90-R	8/10 studies evaluated depressive symptoms. Of the 10 studies, the PEDro scores ranged from 4 to 7.
Lin et al. (28)	2 RCTs, *N* = 39	1) Randomized controlled trials (RCTs) published in a peer-reviewed journal 2) Patients of any age, diagnosed with any type of gynecological cancer (i.e., cancers of the vulva, vagina, cervix, uterus, ovary, fallopian tube and placenta), at any stage of their illness 3) studies including an intervention with any type of exercise component (i.e., aerobic training, muscle strengthening, stretching exercises or education regarding exercise) The mean age of the participants across all studies (7) ranged from 52.1 to 63.9 years.	The length of interventions ranged from 5 days to 6 months. Participants were coached to meet the physical activity guidelines of at least 30 min of physical activity on 5 days per week.	BDI, BDI-II	The mean PEDro score was 5.3 (standard deviation 1.5) out of 10 for the whole analysis (7 studies). 2 of the 7 studies assessed depressive symptoms.
Liu et al. (35)	2 RCTs/CCTs, *N* = 84	1) Patients with CHD (coronary heart disease), regardless of disease stage and severity. Eligible CHD diagnoses included myocardial infarction (MI), angina or a revascularization procedure (coronary artery bypass grafting or percutaneous coronary intervention) 2) Thai Chi Intervention, no limits were imposed on type, duration, frequency, length or intensity of the Tai Chi intervention. 3) RCTs, non-randomized controlled clinical trials (CCTs) 4) Articles published in English or Chinese	Thai Chi groups, duration of 3 months and 2–5 sessions per week.	SDS	2/11 studies evaluated depressive symptoms. The two studies were rated as ‘moderate’ regarding global quality.
Newby et al. (31)	4 RCTs, *N* = 466	1) RCTs 2) Comparison of any intervention to treat depression in patients with prostate cancer 3) Entry criteria for depressive scores did not have to be severe enough to be considered a case of Diagnostic and Statistical Manual major depressive disorder (clinical depression)	For most studies, the intervention was 6–9 weeks. Precise exercise regimen was not stated.	CES-D, HADS, GDS, BDI	4/9 studies evaluated depression. Overall quality was fair (3 studies) to poor (1 study). O'Brien et al. (47)	2 RCTs, *N* = 65	1) RCTs with human participants who were HIV positive 2) adults 18 years of age or older 3) aerobic exercise intervention performed at least three times/week, at least 20 min per session for at least 4 weeks 4) a randomized controlled comparison group	Aerobic exercise was defined as a regimen containing aerobic interventions performed at least three times per week for at least 4 weeks. Aerobic interventions included but were not limited to walking, jogging, cycling, rowing, stair stepping, and swimming. Interventions may or may not have been supervised.	POMS	2/10 studies evaluated depression via POMS and were included in the meta-analysis.
O'Dwyer et al. (44)	3 RCTs, *N* = 75	1) Quasi-randomized and randomized controlled trials in SLE (systemic lupus erythematodes) comparing at least one exercise group to controls 2) Studies evaluating adults diagnosed with SLE by established criteria 3) studies with participants under 18 years of age were excluded 4) studies comparing exercise to no intervention controls, studies comparing different exercise or physical activity protocols (e.g., aerobic exercise vs. strengthening exercise), and studies comparing an exercise-based intervention to another treatment approach (e.g., relaxation)	Exercise-based interventions comprised one or more of the following components: range of motion (stretching), resistance training, or aerobic exercise. 3–6 weeks of duration and 2–3 sessions/week.	BDI	Overall risk of bias of these studies was unclear.
Patsou et al. (21)	14 RCTs, *N* = 1.701	1) Written in English 2) Published in 2011 and beyond 3) Participants were adult women diagnosed with breast cancer based on mammography and biopsy 4) Included an intervention program involving physical activity 5) Randomized controlled trial (experimental) design 5) Results for depression outcomes The median age of the included breast cancer survivors was 52 years. In all studies, women had been diagnosed with 0–IIIc stage breast cancer.	Aerobic, resistance, aerobic and resistance, yoga exercises The length of the interventions ranged from 6 to 52 weeks. The reported exercise frequency was 2–3 sessions per week for the majority of the studies, while duration varied from 30 to 90 min per session and weekly exercise duration ranged from 90 to 270 min. All studies had over 80% up to as high as 99% retention rates, while adherence rates, when reported, varied from 70 to 92.7%.	HADS, CES-D, BDI-II, POMS	The mean PEDro score of the studies was 6.1 ± 2, indicating High quality. Depression was the sole primary outcome measure in only one study, while in three more studies, depression was included either as primary or not as psychosocial/psychological outcome.
Perry et al. (54)	2 RCTs/7 non-RCTs, *N* = 188	1) Participants aged 18 or older and have sustained a TBI (traumatic brain injury) 2) Interventions must utilize physical exercise 3) Depression-related outcome measure	Eight out of the nine studies had an aerobic intervention including treadmill, exercise bike and swimming, one study used a walking intervention. The intervention length was between 8 and 12 weeks, apart from one study which had just two sessions, 1 week apart	BDI, HAM-D, POMS, BRUMS, CES-D, HADS	Majority of included trials was non-randomized, no PEDro scores provided.
Samartzis et al. (36)	9 RCTs (+ 4 RCTs with SSRI comparison), *N* = 3.425	1) An experimental CHF patient group, and a CHF patient group as controls that received standard care 2) Patients were randomly allocated 3) Both groups were evaluated for depression before and after the intervention, by using psychometric instruments 4) data were published	Home and hospital exercise training interventions.	HAM-D,BDI-II, BDI, MADRS, GDS, MAACL	No PEDro scores provided. A trend for superiority of hospital- based exercise training for depression was noted.
Singh et al. (22)	14 RCTs, *N* = 1005	1) RCTs in which at least 50% of the sample was diagnosed with Stage II+ breast cancer 2) Exercise trials 3) Trials were eligible regardless of the level of supervision provided, mode of intervention delivery, intervention duration or intensity. Median recruitment rate was 56% (1–96%), 15 withdrawal rate was 10% (0–41%) and adherence rate was 82% (44–99%).	Aerobic exercise, resistance exercise, combined,	POMS, HADS, CES-D, Greene Climacteric Scale, BDI, Functional Living Index of Cancer	14/61 trials evaluated depression. 38/61 trials were rated as “high quality” according to the PEDro score.
Song et al. (51)	4 RCTs, *N* = 148	1) RCTs and prospective non-randomized controlled and observational studies published in English 2) Parkinson's disease was the primary disease and Tai Chi and/or Qigong were the primary interventions were included. 3) RCTs	3 studies used Thai Chi, 1 study used Qigong. The duration of interventions ranged from 7 to 16 weeks. Individual intervention sessions ranged from 45 to 90 min, and the frequency of classes varied between 1 and 3 times per week.	BDI, GDS, MADRS	5/21 studies (4 RCTs) evaluated depression.
Song et al. (40)	8 RCTs, *N* = 368	1) RCTs 2) patients with ESRD (end-stage renal disease) undergoing HD (hemodialysis) for more than 3 months 3) 18 years and older 4) exercise training compared to routine HD treatment or exercise training with pharmacological treatment compared to the same pharmacological treatment	Aerobic exercise, resistance training, flexibility training, Yoga, Pilates. The duration of one intervention and the total interventions ranged from 15 min to 90 min and 4 weeks to 48 weeks (12 months).	BDI, HADS, Zung Depression Scale	8/15 trials evaluated depression. Cochrane risk of bias tool was applied.
Sosa-Reina et al. (42)	11 RCTs, *N* = 641	1) RCTs comparing types of therapeutic exercise or comparing therapeutic exercise with a control group receiving another intervention or standard care 2) Studies with participants older than 18 years, diagnosed with FMS (fibromyalgia syndrome) in the absence of significant comorbidity 3) Studies using aerobic, strengthening, or stretching exercises or a combination of these were considered. Studies of exercise interventions based on activities such as yoga or tai-chi were excluded. Almost all the participants were women (97.90%). The average age of participants was 42.36 years.	Aerobic exercise, combined exercise, muscle strengthening, flexibility, stretching.	BDI, HAD, and VAS	11/14 studies evaluated depression. It was concluded that all the studies included in this meta-analysis exceeded minimum thresholds for methodological and scientific quality.
Tu et al. (37)	16 RCTs, *N* = 3.226	1) RCTs 2) Included patients (>18 years old) with either systolic HF or HF with a preserved EF of any etiology in the control and in the intervention group (diagnosis based on LVEF or clinical findings) 3) Patients received exercise training either alone or as part of a comprehensive cardiac rehabilitation program (i.e., included components such as psychological intervention or health education) 4) compared with a standard medical treatment or education placebo control group 5) reported the effect of exercise training on depression or depressive Symptoms The median age of the populations ranged between 54 and 81 years.	Walking, bicycle, treadmill, games, jogging, calisthenics, Tai Chi Chuan, and strength training. Frequency 2–7 days/week; duration 20–60 min per session; intensity 40–80% of maximum heart rate, 60–70% of maximal heart rate reserve, or maximum oxygen uptake; and overall length of the programme 6 weeks to 1.5 years.	BDI, CES-D, GDS, HADS, HAM-D, HDCDS	Jadad scale between 2 and 7. Antidepressant effect was not influenced by age, duration of the exercise intervention, or exercise setting, but rather by LVEF and the exercise mode; HF patients with LVEFs of < 50%, as well as aerobic exercise intervention, demonstrated consistent benefits on depressive symptoms.
Vashistha et al. (32)	3 RCTs, *N* = NR	1) RCTs published in English 2) The population of interest was men with clinically diagnosed prostate cancer and participation in a prescribed exercise program 3) Acceptable exercise interventions included Pilates, yoga, mind-body stress reduction, tai chi, walking alone or in combination with cycling, cycling, resistance training, strength training, qigong, aerobic exercise, anaerobic exercise, and/or stretching. 4) Included studies had to contain one of the following primary outcome measures: cancer-specific QOL, overall QOL, fatigue, depression, and/or anxiety. No limit was placed on the duration of exercise intervention or length of follow-up.	Walking, stretching, and light resistance exercises, aerobic exercise, QiGong. Study duration varied from 4 weeks to 6 months (whole study, *N* = 13).	BSI-18	5/13 studies evaluated depression, 3 used the BSI-18 and were enrolled in the meta-analysis. Using the Cochrane Collaboration tool for risk of bias assessment for all 13 RCTs included in our systematic review, we found that 10 studies had high risk of bias, two had unclear risk of bias, and one had low risk of bias. Wang et al. (34)	4 RCTs, *N* = 245	1) Published RCTs 2) Patients with CVD including ischemic heart disease or coronary artery disease, cerebrovascular disease, diseases of the aorta and arteries, and peripheral vascular disease 3) Articles that compared an intervention group (e.g., tai chi, qigong, baduanjin) with a control group that performed other exercises (egg, strength exercises), that received usual care, or that did not undergo any intervention.	Thai Chi, aerobic exercise. Duration of the intervention between 5 and 12 weeks.	HAMD, POMS	4/35 studies evaluated depression. A low risk of incomplete outcome bias was reported in 31 articles (88.5%), whereas a low risk of selective reporting bias was reported in most articles (*n* = 28, 73.6%).
Wayne et al. (30)	7 RCTs, *N* = 783	1) Randomized controlled trials (RCTs), prospective non-randomized controlled studies, and prospective non-controlled studies published in English 2) cancer was the primary disease and Tai Chi and/or Qigong were the primary interventions Most studies evaluated breast cancer patients. Other cancers included ovary, lung, gastrointestinal and colorectal cancer.	Intervention duration between 6 and 12 weeks.	POMS, CES-D, BDI, HADS, BSI-18, DASS-21	7/15 studies evaluated depression. Quality of RCTs indicated an overall low to high risk of bias For the 7 RCTs.
Ying et al. (33)	2 RCTs, *N* = 124	1) All participants were adult men and the diagnoses of prostate cancer were based on pathology reports and staging studies 2) The participants all underwent ADT (androgen deprivation therapy) for at least 3 months 3) Lifestyle interventions contain dietary advices, aerobic and resistance exercises, and physical activity 4) The patients had minimum lifestyle intervention duration of 6 weeks Exclusion criteria: Men with diabetes, uncontrolled hypertension, cardiovascular disease and unstable bony metastases.	Aerobic and resistance exercise. Duration between 12 and 24 weeks.	NR	Jadad scores between 3 and 5. 2/11 RCTs evaluated depression.
Zhu et al. (23)	8 RCTs, *N* = 751	1) English language 2) Adopted a randomized controlled trial design, comparing exercise intervention group with control group (usual care, maintain current activity level, or waitlist) 3) Included adults diagnosed with breast cancer 4) Evaluated the effects of exercise in breast cancer patients.	The main types of exercise interventions reported in this meta-analysis were aerobic, resistance, and stretching exercises. Duration in the whole study (*n* = 33) between 5 weeks and 6 months.	NR	8/33 studies evaluated depression. Delphi criteria were used for assessment of quality, the studies were heterogenous in the level of quality.
Zhou et al. (45)	3 RCTs, *N* = 128	1) participants with a diagnosis of AL [either acute myelocytic leukemia (AML) or acute lymphoblastic leukemia (ALL)] undergoing induction therapy or post-remission therapy 2) Intervention: an exercise component, regardless of the type of exercise 3) Comparison intervention: standard care with no exercise intervention or instruction 4) Outcome measures including depression 5) Study design: a randomized controlled trial (RCT) or a quasi-experimental design trial	Aerobic exercise, mixed-modality exercise. Duration was reported for 2 of the studies with 3–12 weeks.	HADS	3/9 studies evaluated depression. Risk of bias was heterogenous.

*BDI, Beck Depression Inventory; CDI, Children Depression Inventory; CES-D, Center for Epidemiological Studies Depression Scale; DASS, Depression Anxiety Stress Scales; GDS, Geriatric Depression Scale); HAD, Hospital Anxiety and Depression Scale; HAM-D, Hamilton Rating Scale for Depression; SCL-90, Symptom Checklist-90; TAS, Toronto Attitude Scale; POMS, Profile of Mood States; SAS, Symptom Assessment Scale; CSDD, Cornell Scale for Depression in Dementia; IDS-SR, Inventory of Depressive Symptomatology Self Report; LPD, Levine-Pilowsky Depression; MADRS, Montgomery-Asberg Depression Rating Scale; MDI, Major Depression Inventory; POMS, Profile of Mood States; QOL, Quality of Life; DASS, Depression Anxiety Stress Scales; TAS, Toronto Attitude Scale; MAACL, Multiple Affect Adjective Checklist; HDCDS, Hare-Davis Cardiac Depression Scale; SDS, Self-rating Depression Scale; BSI-18, Brief Symptom Inventory; BRUMS, Brunel Mood Scale; NR, not reported*.

**Table 3:** Outcomes of the included meta-analyses.

**References**	**Results/Main Outcomes**
Adamson et al. (50)	Results from the meta-analysis yielded a small but statistically significant effect of 0.28 for depression reduction overall (SE = 0.07; 95% CI: 0.15 to 0.41; *p* = 0.00). The effect of exercise on depression is slightly larger (0.36) in the MS studies compared with the other studies (0.20), but this difference was not statistically significant.
Bergenthal et al. (46)	The pooled result of three trials (*N* = 249) for depression shows a statistically significant benefit for the exercise arm (SMD = 0.25; 95% CI:−0.00 to 0.50; *p* = 0.05).
Brown et al. (24)	Exercise provided a small overall reduction in depressive symptoms compared to standard care among all types of cancer ds = −0.13, 95% CI: −0.26 to −0.01). Collectively, the 40 effect sizes lacked homogeneity *I*^2^ = 55%, 95% CI: 35 to 68, *p* < 0.001. Subgroup analysis by cancer type revealed significant reductions in depressive symptoms among breast cancer survivors *d* = −0.17, 95% CI: −0.32 to −0.02, but no significant difference in depressive symptoms among prostate, leukemia, lymphoma, and colorectal cancer survivors.
Buffart et al. (25)	After excluding outliers, yoga resulted in significant large reductions in depression (*d* = −0.69; 95% CI: 1.02 to −0.37).
Carayol et al. (17)	Exercise resulted in an improvement of depressive symptoms (*d* = −0.275, 95% CI: −0.457 to −0.094, *p* = 0.003). *I*^2^ = 39% (*p* = 0.09).
Chung et al. (38)	Exercise improved depressive symptoms (*d* = −1.233, SE = 0.482, 95% CI: −2.177 to −0.289, *p* = 0.010). Heterogeneity: χ^2^ = 23.80,*p* < 0.001; *I*^2^ = 87.40%
Craft et al. (26)	Mean ES of (−0.22, 95% CI: −0.43 to −0.009; *p* = 0.04) under a random effects model, when comparing exercise interventions to control groups.
Cramer et al. (19)	Evidence for large short-term effects were found for depression (SMD = −1.59, 95% CI: −2.68 to −0.51; *p* < 0.01). Heterogeneity: Chi^2^ = 84.03, df = 5 (*p* < 0.00001); *I*^2^ = 94% Long-term effects (2 studies, *N* = 43) of up to 24 weeks did not show significant results.
Cramer et al. (18)	Comparison of yoga vs. no therapy: Yoga did not appear to reduce depression (pooled SMD = −0.13, 95% CI −0.31 to 0.05; seven studies, 496 participants; low-quality evidence). Comparison of yoga vs. psychosocial/educational interventions provided moderate-quality evidence indicating that yoga can reduce depression (pooled SMD = −2.29, 95% CI: −3.97 to −0.61; 4 studies, 226 participants).
Dalgas et al. (48)	In summary the meta-analysis indicated a small beneficial effect of exercise on depressive symptoms in people with MS. The SMD across studies was(*g* = −0.37, 95% CI: −0.56 to −0.17).
Eng and Reime (52)	Overall, physical exercise resulted in less depressive symptoms over 13 studies involving 1022 patients (SMD = −0.13, 95% CI: −0.26 to −0.01, *I*^2^ = 6%, *p* = 0.03) with low heterogeneity. Ten studies evaluated the effect on depressive symptoms after a period of time had elapsed following the exercise sessions (range from 10 weeks to 9 months). Physical exercise did not change depressive symptoms over these 10 follow-up studies involving 889 patients (SMD = −0.04, 95% CI: −0.17 to 0.09, *I*^2^ = 1%, *p* = 0.53).
Ensari et al. (49)	The weighted mean ES was small, but statistically significant (*g* = 0.36, SE = 0.09, 95% CI: 0.18 to 0.54, *Z* = 3.92, *p* < 0.001), indicating the exercise training resulted in an improvement in depressive symptoms compared to control.
Fong et al. (27)	Measured by the Beck depression inventory, physical activity was associated with reduced depression (−4.1, 95% CI: −6.5 to −1.8; *p* < 0.01) in survivors of mixed types of cancer. Four other studies in the sample used the HADS (two) or POMS (two). The results for HADS were (−0.5, 95%CI: −2.8 to 1.7, *p* = 0.64 and for POMS −7.5, 95 CI:−16.0 to 1.0, *p* = 0.09).
Furmaniak et al. (20)	Exercise may lead to little or no improvement in depression (SMD = −0.15, 95% CI: −0.30 to 0.01, test for overall effect: *Z* = 1.86; *p* = 0.062). Heterogeneity: Tau^2^ = 0.0; Chi^2^ = 3.73, df = 5; *p* = 0.59; *I*^2^ = 0.0%.
Gomes Neto et al. (39)	Exercise lead to a reduction in depression symptoms (−7.32; 95% CI: −9.31 to −5.33). Test for overall effect size *Z* = 7.21, *p* < 0.00001.
Graven et al. (53)	When the data from the two studies was pooled; (SMD = −2.03, 95% CI: −3.22 to −0.85) immediately after the intervention phase (note, different time points were used in the two studies for the follow-up assessment).
Herring et al. (16)	Exercise training significantly reduced depressive symptoms by a heterogeneous mean effect of 0.55 (95% CI: 0.31 to 0.78, *p* < 0.001). A significant improvement in fatigue (β = 0.37, Z = 2.21, p ≤ 0.03) accounted for significant variation in the overall effect of exercise on depressive symptoms.
Langhorst et al. (41)	Meditative movement therapies improved depressive symptoms (SMD = −0.49, 95% CI: −0.76 to −0.22, *p* = 0.0004 Heterogeneity *I*^2^ = 27%; Tau^2^ = 0.03). 2 studies evaluated the follow-up (*N* = 132), with no significant improvement of depressive symptoms. In subgroup analyses, only Yoga yielded significant effects on depression at final treatment.
Liang et al. (43)	A statistically significant difference was observed (MD = −2.31, 95% CI: −3.33 to −1.30, p = 0.001), which indicated that home-based exercise interventionsreduced the depression scores, compared to the control groups. No significant heterogeneity between home-based exercise groups and control groups (*p* = 0.24, *I*^2^ = 30%).
Lin et al. (29)	Improvement of depressive symptoms (−0.95, 95% CI: −1.55 to −0.36, test for overall effect: *Z* = 3.15, *p* = 0.002). Heterogeneity: τ^2^ = 0.63, χ^2^ = 66.81, df = 7, *p* < 0.00001); *I*^2^ = 90%.
Lin et al. (28)	No significant effects were found for depression and health-related quality of life. (No SMD or CI provided) Liu et al. (35)	The Thai Chi group had a significantly lower level of depression (SMD 9.42, 95% CI: 13.59 to 5.26, *p* < 0.001, *I*^2^ = 81%, *N* = 168) compared with the non-active control groups.
Newby et al. (31)	Exercise interventions significantly reduced depressive symptoms (Point estimate −0.961, SE = 0.319, CI 95% −1.585 to −0.337, *Z* = −3.017, *p* = 0.003).
O'Brien et al. (47)	One meta-analysis was performed and demonstrated a significant improvement in the depression-dejection subscale of the Profile of Mood States Scale (POMS) by a reduction of 7.68 points for participants in the aerobic exercise intervention group compared with the non-exercising control group (95% CI: −13.47 to −1.90, *p* = 0.009, *I*^2^ = 94%, *p* < 0.0001).
O'Dwyer et al. (44)	A meta-analysis including three studies found significantly lower depression scores in the exercise groups compared to controls (SMD = −0.40 SD; 95% CI: −0.71 to −0.09, test for overall effect size *Z* = 2.54, *p* = 0.01).
Patsou et al. (21)	Reduction in depressive symptoms showed a small to moderate effect of depressive symptoms in favor of the exercise *g* = −0.38 (95% CI −0.89 to 0.13, *p* = 0.14). With regard to the type of the exercise intervention, aerobic interventions yielded a large and significant effect on depression at the last follow-up (3–6 months) measurement compared with the control groups (*g* = −1.23, 95% CI: −1.97 to −0.49, *p* = 0.001).
Perry et al. (54)	This represents a statistically significant, positive small to medium overall effect size of physical exercise to reduce depressive symptoms in people following TBI (SMC = 0.48, 95% CI = 0.16 to 0.81). Tests of heterogeneity were significant (*p* < 0.01), confirming that heterogeneity was present amongst the studies included in the analysis.
Samartzis et al. (36)	Interventions using exercise training appeared more effective compared to usual care (SMD = 0.391, 95% CI: 0.213 to 0.569). There was a trend for SSRI superiority compared to exercise training for improving depression (*Q* = 3.257, df = 1, *p* = 0.071).
Singh et al. (22)	Large effect in favor of exercise SMD = 0.66, 95%, CI: 0.52 to 0.80, *p* < 0.01, *I*^2^ = 90%; high heterogeneity. Intervention duration had an effect on depression (χ^2^ = 7.93, df = 1, *p* < 0.01), with interventions lasting longer than 12 weeks producing a large effect (SMD = 0 .84, 95% CI: 0.65 to 1.03, *p* < 0.01) and interventions lasting 12 weeks or less having a moderate effect (SMD = 0.44, 95% CI: 0.23 to 0.65, *p* < 0.01).
Song et al. (51)	A fixed-effect model indicated that TCQ significantly reduced depression scores compared to control groups, with an overall medium effect size (*g* = −0.457, 95% CI: −0.795 to −0.118, *p* = 0.008). *Q*- value (*p* = 0.739) and *I*^2^ (0%) indicated limited heterogeneity.
Song et al. (40)	Exercise training was able to reduce depression in HD patients (SMD = -0.95, 95% CI: −1.18 to −0.73; *Z* = 8.33, *p* < 0.00001).
Sosa-Reina et al. (42)	There is strong evidence from intention-to-treat and per protocol analysis that exercise reduces symptoms of depression (−0.40, 95% CI: −0.55 to −0.24; *p* < 0.001). Values of Cohen's g suggested that exercise had a small effect on symptoms of depression.
Tu et al. (37)	Strong evidence of a decrease in the symptoms of depression with exercise (SMD −0.38, 95% CI: −0.55 to −0.21, *p* < 0.00001) in 3–6 months follow-up.
Vashistha et al. (32)	The pooled data did not reveal a significant improvement in depression (−3.02, 95%CI: −7.83 to 1.79, test for overall effect: *Z* = 1.23, *p* = 0.22). Heterogeneity: Tau^2^ = 9.80; Chi^2^ = 4.64, df = 1, *p* = 0.03; *I*^2^ = 78%.
Wang et al. (34)	The HAMD scores of patients performing TCEs improved (MD −3.97, 95% CI: −5.05 to −2.89, *p* < 0.001; *I*^2^ = 0, *p* = 0.91) compared with those of patients in the control group, based on a random-effects model. The POMS depression scale scores of the patients performing TCEs significantly improved (MD −3.02, 95% CI: −3.50 to −2.53, *p* < 0.001; *I*^2^ = 0%, *p* = 0.76) compared with those of patients in the control group, based on a random-effects model.
Wayne et al. (30)	The overall effect size based on a random-effects model favors TCQ on depression in cancer patients (*g* = −0.27, 95% CI: −0.44 to −0.11, *p* = 0.001). A subgroup meta-analysis limited to five RCTs using an active control group showed a statistically non-significant trend toward TCQ improving depression (*g* = −0.22, 95% CI:−0.47 to 0.02, *p* = 0.080). A subgroup meta-analysis limited to the three RCTs with a no-treatment control group showed a statistically positive effect of TCQ.
Ying et al. (33)	No obvious difference in mitigating depression (SMD = −0.18, 95% CI: −0.54 to 0.17, *p* = 0.31).
Zhu et al. (23)	Exercise intervention reduced depression, (SMD = −2.08, 95% CI: −3.36 to −0.80, *p* = 0.001, *I*^2^ = 2%, *p* = 0.41).
Zhou et al. (45)	Based on the data for depression, there were no significant differences in these parameters between the exercise and control groups (SMD = −0.15, 95% CI: −0.51 to 0.22, *p* = 0.28, *p* for heterogeneity = 0.57, *I*^2^ = 0%).

#### Exercise Interventions in Cancer Patients

##### Exercise interventions in breast cancer patients

Our search resulted in seven meta-analyses that met the inclusion criteria. The majority found positive effects of exercise on depressive symptoms in these cohorts (17, 21–23, 55). Different types of interventions were summarized (aerobic, resistance, aerobic and resistance, yoga exercises), with one meta-analysis providing more detailed information about differences between the exercise types: aerobic interventions yielded a large and significant effect on depression at the last follow-up measurement compared with the non-exercising control group (21). Another meta-analysis pointed toward the beneficial effects of an intervention duration of longer than 12 weeks (22).

One meta-analysis only showed little improvement in depression after exercise intervention (20). The two remaining meta-analyses with overlapping populations of Cramer et al. (18, 19) were heterogeneous. In the 2012 analysis, large short-term effects were shown (19), in the 2017 analysis these results could not be reproduced (18).

##### Exercise interventions in mixed cancer-samples

Most of these meta-analyses included a majority of breast-cancer patients.

*Yoga intervention* Buffart et al. showed significant large reductions in depressive symptoms of yoga interventions, one of the included RCTs included lymphoma patients, the other 6 trials breast cancer (25). Lin et al. (29) summarized 6/8 RCTs with breast cancer and different forms of yoga interventions and showed that depressive symptoms improved significantly in the intervention groups (29).

In the cohort of Wayne et al. breast cancer was also the main disease. The overall effect size favored Thai Chi and QiGong interventions on depressive symptoms (30).

*Aerobic or mixed intervention* Brown et al. (24) (65% of patients with breast cancer) found a small overall reduction in depressive symptoms of aerobic and yoga interventions compared to standard care among all types of cancer. Subgroup analysis by cancer type revealed significant reductions in depressive symptoms among breast cancer survivors, but no significant difference in depressive symptoms among prostate, leukemia, lymphoma, and colorectal cancer survivors.

Another meta-analysis by Craft et al. revealed modest beneficial effects of exercise (26). Fong et al. evaluated 75% breast cancer patients and showed different outcomes with regard to different depression scales: measured by the BDI, physical activity (aerobic) was associated with reduced depression. Subgroups of four RCTs used the HAMD and POMS scales, the outcomes in this cohort were not significant (27).

In another meta-analysis by Lin et al. (28) with various gynecological cancer-types and various types of activity (i.e., aerobic training, muscle strengthening, stretching exercises or education regarding exercise) no statistically significant improvement could be shown. None of the included RCTs appeared in both analyses (28).

##### Exercise interventions in Patients with Prostate Cancer

Three meta-analyses were found eligible for this review with regard to prostate cancer. All of them included various types of exercise and one meta-analysis included lifestyle interventions consisting of exercise and other interventions (e.g., dietary advice) (33). The largest meta-analysis with four RCTs found significantly reduced depressive symptoms, while the two smaller ones with 3 RCTs and 2 RCTs found no statistically significant differences (31–33).

#### Exercise Interventions in Patients With Cardiovascular Disease and Coronary Heart Disease

For cardiovascular diseases, one meta-analysis could be included in our meta- review (34). 4 RCTs summarizing patients with ischemic heart disease or coronary artery disease, cerebrovascular disease, diseases of the aorta and arteries, and peripheral vascular disease compared Thai Chi, qigong, baduanjin interventions to control interventions (e.g., strength training or no intervention). Both outcome measures (HAMD and POMS) showed significant improvements in the intervention groups. One meta-analysis with 2 RCTs/CCTs analyzed the effects of 3-months of Thai Chi Interventions and found positive effects in the intervention group concerning the depressive symptoms (35).

#### Exercise Interventions in Patients With Heart Failure

The two included meta-analyses showed an improvement in depressive symptoms compared to standard care in large cohorts (36, 37).

#### Intradialytic Exercise Interventions and Exercise Interventions in Hemodialysis Patients

We could include two meta-analyses for the cohort of intradialytic exercise (38, 39). Exercise interventions included aerobic training, resistance training, or a combination with strength training or range of motion. Both publications showed significant improvements of depressive symptoms (38, 39).

One meta-analysis included intradialytic exercise patients and exercise interventions in hemodialysis patients on non-dialytic days (40). Beneficial effects of exercise were reported.

#### Exercise Interventions in Patients With Fibromyalgia, Ankylosing Spondylitis and Lupus Erythematodes (LE)

The two cited meta-analyses for fibromyalgia (41, 42) were able to present improved depressive symptoms. The interventions were heterogeneous with Tai Chi and/or Yoga, and/or Qi Gong (41) and aerobic exercise, combined exercise, muscle strengthening, flexibility, stretching (42).

Our search resulted in one meta-analysis that revealed a statistically significant beneficial effect of home-based stretching exercise in reducing depressive symptoms in patients with ankylosing spondylitis (43).

In a meta-analysis of patients with LE, significantly lower depression scores were found in the exercise groups (stretching or aerobic exercise) compared to controls (44).

#### Exercise Interventions in Patients With Acute Leukemia and Other Hematological Malignancies

Our systematic search resulted in two meta-analyses, one including solely acute leukemia (45) and one including various hematological malignancies (46). Zhou et al. found no significant differences between the exercise (aerobic exercise, mixed-modality exercise) and control groups, whereas Bergenthal showed a statistically significant benefit for the exercise group with aerobic exercise or combined aerobic/strength exercise in a larger cohort.

#### Exercise Interventions in Patients With HIV

We were able to identify one meta-analysis with a small sample size and various forms of exercise interventions (e.g., walking, jogging, cycling, rowing, stair stepping, and swimming). A significant improvement could be displayed in the depression-dejection sub scale of the POMS d (47).

#### Exercise Interventions in Patients With Neurological Diseases

Three meta-analyses revealed small but significantly positive effects of exercise on depressive symptoms in multiple sclerosis (16, 48, 49). Different forms of exercise were allowed (Aerobic, Resistance Aerobic+, Yoga).

In Parkinson's disease, exercise interventions (Tai Chi and/or Qigong) led to significantly improved depressive symptoms (51).

Overall, physical exercise resulted in less depressive symptoms in the two suitable meta-analyses of stroke patients (52, 53). After 10 weeks of follow-up, this beneficial effect could not be maintained (52).

One meta-analysis included various neurological disorders and various exercise interventions. Depressive symptoms were significantly reduced. The effect of exercise on depression was slightly larger in the MS studies compared with the other studies. In addition to the above mentioned neurological disorders (with overlapping single studies), other diseases like Alzheimer's disease, were included (50).

#### Exercise Interventions in Patients With Traumatic Brain Injury

In one meta-analysis, two RCTs and seven non-RCTs were included and found statistically significant, positive effects of exercise on depressive symptoms (54).

## Discussion

We were able to include 39 meta-analyses confirming that the application of exercise as an add-on treatment in patients with somatic disorders is a frequently studied intervention. As stated above, different reasons for this observation can be discussed: the positive effects in depression alone (15), the principle lack of side-effects and the overall improvement of fitness and mortality. In our meta-review, most meta-analyses showed these (expected) beneficial effects of exercise on depressive symptoms (33/39). The six meta-analyses without these effects evaluated different exercise modalities in different types of diseases (18, 20, 28, 32, 33, 45). One of these meta-analyses provided data of breast cancer patients receiving yoga therapy and found no significant effect on depressive symptoms of yoga when comparing to no therapy, but did find significant improvements of depressive symptoms when comparing yoga to psychosocial intervention (7 and 4 included trials) (18). Another meta-analysis with breast-cancer patients found no significant results (20). The smallest of the included meta-analyses with 39 participants (2 trials) also evaluated depressive symptoms in any type of gynecological cancer and found no significant effects (28). Neither of the two meta-analyses with prostate cancer patients nor the meta-analyses with acute lymphoblastic leukemia (ALL) patients found significant reductions in depressive symptoms (32, 33, 45).

Five meta-analyses reported long-term effects with follow-up periods from 3 to 9 months of the exercise interventions (19, 21, 37, 41, 52). The results were heterogeneous with two meta-analyses showing no significant effects in a 3 to 9 months period (19, 52). One meta-analysis only found positive long-term effects during 3 to 6 months for one of the possible exercise interventions in subgroup analyses (41). The two remaining meta-analyses pointed to positive long-term effects in a 3–6 months period (21, 37). In all of these studies except one (37) the follow-up cohort was much smaller compared to the overall sample size. The small sample sizes on the one hand and the small number of meta-analyses in total restrict the generalizability of these results. Future studies should include a follow-up period of at least 3 months (preferably three and 3 months assessments) to accumulate data for meaningful statements.

Following the American College of Sports Medicine (ACSM) guidelines, healthy adults should engage in moderate aerobic exercise training for ≥30 min per day on ≥5 days per week, vigorous aerobic exercise training for ≥20 min per day on ≥3 days per week, or a combination of both moderate and vigorous aerobic training. On another 2–3 days, healthy adults should perform resistance exercises for each of the major muscle groups and flexibility exercises for each major muscle-tendon groups on ≥2 days per week (56). Future studies should lean on these recommendations to help homogenize the results.

In literature, many different rating scales (self- and observer-rating) have been introduced to assess depressive symptoms, reflecting the variety of questionnaires used in the original trials and the consecutive meta-analyses. For example, the practice guidelines for the treatment of depression in Germany (57) list nine possible questionnaires as valid tools. In specific somatic disorders, validation studies of the most often used scales were performed: HAMD for multiple sclerosis patient (58) and for stroke patients (59). BDI for cancer patients (60) and heart failure patients (61). In a mixed cancer population, a review recommended the CES-D as most precise instrument if depression is the sole focus (62). It has been stated that discrepancies between BDI and HAMD scores (self- and observer rating scales) could be due to different personality traits (e.g., high neuroticism is associated with higher BDI scores) and that therefore both should be regarded separately (63). To date, valid recommendations for all included somatic disorders or general recommendations for specific questionnaires to access depressive symptoms could not be identified. For future studies, similar outcome measured should be implemented to ensure comparability and both self- and observer rating scales should be combined.

The possible reasons for the varying observations regarding the different intervention types and the measuring time points could be the same reasons that complicate meta-analyses of the existing exercise trials. As stated above, in none of the meta-analyses, the included trials had matching exercise protocols regarding type, duration and frequency of the intervention. Most often no data was provided about the attendance rate of the participants and about the aerobic capacity before/after the intervention as a controlling variable. The quality of the trials ranged from very low to good quality (also with regard to the exercise-specific difficulty of blinding the intervention). In none of the meta-analyses the depressive symptoms after the exercise intervention were the primary endpoint in all of the included trials, so moreover the validity of the conclusions has to be discussed cautiously.

In summary, we were able to identify a large interest in this field of research for the above demonstrated reasons. In most of the meta-analyses, regardless of the underlying somatic disease, beneficial effects of exercise on depressive symptoms could be observed—keeping in mind that depressive symptoms were mainly secondary outcomes in the included studies. Some of the somatic diseases, especially cancer and in this field breast cancer, were overrepresented compared to others like heart failure.

More standardized trials with better comparability are required to draw specific conclusions about the recommended type and duration of exercise in different diseases. The current findings point to beneficial effects of various forms of exercise, but the implementation in guidelines and for example in therapy strategies that are supported more extensively is still difficult because of the lack of clarity of specific outcomes. Especially the diversity of outcome parameters impedes the comparability of interventions within and between somatic disorders. Moreover, most meta-analyses did not report types of other antidepressant interventions like medication or psychotherapy. Thus, it is difficult to answer the question whether exercise therapy is efficient as an add-on treatment to an ongoing pharmaco- or psychotherapy or whether it should be offered as a single intervention in patients with somatic disorders and depression. Finally, information is sparse regarding whether patients continue to exercise after the intervention (e.g., in sport clubs) or whether their overall activity level (e.g., measured by pedometers or more accurately by accelerometers) increases and how this is related to outcome.

## Conclusion

In this meta-review, we provide an overview of existing evidence for the effects of exercise on depressive symptoms in various somatic disorders. The results are promising, but meaningful recommendations are lacking because of heterogeneous study protocols. For better comparability, we would recommend to implement the following standards in future studies: homogeneous outcome measures, one self-rating scale (e.g., BDI) and one observer-rating scale (e.g., HAMD or MADRS). Moreover, specific information of other antidepressant treatments (especially medication and psychotherapy) should be consistently reported. State and duration of the somatic disease should be provided. Future studies should lean on recommendation of the ACSM regarding type and intensity of the intervention (e.g., moderate aerobic exercise training for ≥30 min per day on ≥5 days per week or vigorous aerobic exercise training for ≥20 min per day on ≥3 days per week; on another 2–3 days, resistance exercises for each of the major muscle groups and flexibility exercises for each major muscle-tendon groups on ≥2 days per week) (56). For aerobic training, bicycle ergometers are a widely-used and practical possibility (also for e.g., patients with arthrosis). The duration of the intervention should last at least 12 weeks. Attendance rates, setting (e.g., group activity, with/without supervision) and training intensity should be monitored, preferably with spiroergometric examinations at the start and the end of the study. Follow-up examinations of 3 and 6 months should be performed, and also in this period, information about antidepressant medication should be provided. On these occasions, information about the overall fitness level (e.g., via pedometer/accelerometer measurements over at least 3 days or via activity questionnaires like IPAQ) should be documented.

## Author Contributions

AR and AH conceived the study. AR and SK performed the qualitative analyses. AR, SK, IM, and AH wrote the first draft. AS and PF supervised the project. BM provided methodological advice; and all authors were involved in the reviewing the manuscript and approved the final version of the manuscript.

### Conflict of Interest Statement

The authors declare that the research was conducted in the absence of any commercial or financial relationships that could be construed as a potential conflict of interest.
